# Supplementary material for: Diversity trapped in cages: Revision of Blumenavia Möller (Clathraceae, Basidiomycota) reveals three hidden species
Source: PLoS One. 2020 May 1;15(5):e0232467. doi: 10.1371/journal.pone.0232467 (PMC7194408; doi:10.1371/journal.pone.0232467)
Supplement: Attachment — Submitted filename: Response to Reviewers.docx [file pone.0232467.s002.docx]

**PONE-D-20-01494R1- Response to Reviewers**

**Diversity trapped in cages: revision of *Blumenavia* Möller (Clathraceae, Basidiomycota) reveals three hidden species**

Gislaine C. S. Melanda et al.

**Editorial Decision**

Thank you for submitting your manuscript to PLOS ONE. After careful consideration, we feel that it has merit but does not fully meet PLOS ONE’s publication criteria as it currently stands. Therefore, we invite you to submit a revised version of the manuscript that addresses the points raised during the review process.

===

All three reviewers found your study interesting. However, they also suggested revisions. Although overall it is a minor revision, they are many places that the authors have to pay close attention. Especially, the authors need to respond carefully to the reviewers 1 and 3 as they requested a thorough edition.

We would appreciate receiving your revised manuscript by Apr 11 2020 11:59PM. When you are ready to submit your revision, log on to https://www.editorialmanager.com/pone/ and select the 'Submissions Needing Revision' folder to locate your manuscript file.

If you would like to make changes to your financial disclosure, please include your updated statement in your cover letter.

To enhance the reproducibility of your results, we recommend that if applicable you deposit your laboratory protocols in protocols.io, where a protocol can be assigned its own identifier (DOI) such that it can be cited independently in the future. For instructions see: http://journals.plos.org/plosone/s/submission-guidelines#loc-laboratory-protocols

Dear editor, thank you very much for giving us the opportunity to send a revised manuscript. Thank you also to Reviewers for some of his/her constructive comments.

**Journal Requirements**:

1. Please ensure that your manuscript meets PLOS ONE's style requirements, including those for file naming. The PLOS ONE style templates can be found at

http://www.journals.plos.org/plosone/s/file?id=wjVg/PLOSOne_formatting_sample_main_body.pdf and

http://www.journals.plos.org/plosone/s/file?id=ba62/PLOSOne_formatting_sample_title_authors_affiliations.pdf

Authors: We’ve checked all guidelines and verified the correction of any mistake.

2. In your Methods section, please provide additional details regarding the samples used in your study and ensure you have described the source. For more information regarding PLOS' policy on materials sharing and reporting, see https://journals.plos.org/plosone/s/materials-and-software-sharing#loc-sharing-materials.

Authors: The text was modified to better explanation. All herbaria gave the permission from the analysis of the exsiccates, and the authorization for collecting material were obtained.

3. Please take this opportunity to be sure you have met all of our guidelines for new species. When publishing papers that describe a new fungal taxon name, PLOS aims to comply with the requirements of the International Code of Nomenclature for algae, fungi, and plants (ICN). The following guidelines for publication in an online-only journal have been agreed such that any scientific fungal name published by us is considered effectively published under the rules of the Code. Please note that these guidelines differ from those for zoological nomenclature.

Effective January 2012, "the description or diagnosis required for valid publication of the name of a new taxon" can be in either Latin or English. This does not affect the requirements for scientific names, which are still to be Latin. Also effective January 2012, the electronic PDF represents a published work according to the ICN for algae, fungi, and plants. Therefore the new names contained in the electronic publication of a PLOS ONE article are effectively published under that Code from the electronic edition alone, so there is no longer any need to provide printed copies.

For proper registration of the new taxon, we require two specific statements to be included in your manuscript.

a) In the Results section, the globally unique identifier (GUID), currently in the form of a Life Science Identifier (LSID), should be listed under the new species name, for example: Hymenogaster huthii. Stielow et al. 2010, sp. nov. [urn:lsid:indexfungorum.org:names:518624]

You will need to contact either Mycobank or Index Fungorum to obtain the GUID (LSID).

Authors: All the new species described in the manuscript have Mycobank number; the include diagnosis and selection of type as required by the updated *International Code of Nomenclature for algae, fungi, and plants* (ICN).

b) In the Methods section, include a sub-section called "Nomenclature" using the following wording (this example is for taxon names submitted to MycoBank; please substitute appropriately if you have submitted to Index Fungorum and use the prefix http://www.indexfungorum.org/Names/NamesRecord.asp?RecordID= ): The electronic version of this article in Portable Document Format (PDF) in a work with an ISSN or ISBN will represent a published work according to the International Code of Nomenclature for algae, fungi, and plants, and hence the new names contained in the electronic publication of a PLOS ONE article are effectively published under that Code from the electronic edition alone, so there is no longer any need to provide printed copies.

In addition, new names contained in this work have been submitted to MycoBank from where they will be made available to the Global Names Index. The unique MycoBank number can be resolved and the associated information viewed through any standard web browser by appending the MycoBank number contained in this publication to the prefix http://www.mycobank.org/MB/. The online version of this work is archived and available from the following digital repositories: [INSERT NAMES OF DIGITAL REPOSITORIES WHERE ACCEPTED MANUSCRIPT WILL BE SUBMITTED (PubMed Central, LOCKSS etc)].

All PLOS ONE articles are deposited in PubMed Central and LOCKSS. If your institute, or those of your co-authors, has its own repository, we recommend that you also deposit the published online article there and include the name in your

article.

A complete explanation of our guidelines for publishing new species can be found on our website: http://www.plosone.org/static/guidelines#fungal

Authors: A sub-section called "Nomenclature” was included in the Method section.

**Review Comments to the Author**

**Reviewer #1:** The authors have done a very thorough revision of *Blumenavia* genus, including type specimens. This is a poorly studied group with rare species mainly because of their receptacles ephemeral and fragile. The manuscript presents original data and relevant information. Some modification are necessary to in order to render it acceptable for publication.

My suggestions are included in the attached PDF document.

Authors: We are thankful for your contribution. All the minor errors to change words were accepted and corrected in the text.

**Reviewer #1:** Mao-Qiang He et al. 2019. Notes, outline and divergence times of Basidiomycota. Fungal Diversity 99:105–367. They only consider Phallaceae. Published December 2019.

Authors: We adopted the classification of Hosaka et al. 2006 (Molecular phylogenetics of the gomphoid-phalloid fungi with an establishment of the new subclass Phallomycetidae and two new orders. Mycologia, 98(6), 2006, pp. 949–959) who bring a phylogenetic well supported clades of six Families in Phallales, and Cabral et al. 2012 (*Abrachium*, a new genus in the Clathraceae, and *Itajahya* reassessed. Mycotaxon -Ithaca Ny- 119(1):419-429) who confirm the phylogeny of Clathraceae segregated of Phallaceae, as well as made an amendment to it. In this article mentioned for the reviewer (Mao-Qiang He et al. 2019), they used sequences of: *Lysurus borealis, Aseroë rubra, Clathrus chrysomycelinus, Lysurus mokusin, Mutinus elegans* and *Phallus hadriani*, but they didn’t recognize the Clathraceae in their discussion and didn’t explain why. As we can see in zoom of Fig. 2 for Mao-Qiang He et al. 2019 (image printed below), two clades were formed: one with *Aseroë rubra* and *Clathrus chrysomycelinus* (Clathraceae, yellow arrow), and the other with members of Phallaceae, *Lysurus borealis, Lysurus mokusin, Mutinus elegans* and *Phallus hadriani* (red arrow), this can confirm the existence of these two families.

**Reviewer #1:** Line 75: “However, despite the proposed synonymy, the author did not analyze the type specimen of B. usambarensis, from Tanzania, but only exsiccates from Brazil and Trinidad and Tobago.”

to add in the end of the phrase: as *B. angolensis*.

Authors: The suggestion was not accepted, because Dring's analyzed specimens were collected and determined as *Blumenavia usambarensis*: one from São Paulo and the other from Trinidad, only in his work he determined it as *B. angolensis*.

**Reviewer #1:** Line 84: Geografical distribution: It was not discussed in the article, it only presents a figure (Fig 3).

What is the relevance of geographical distribution to delimit species in the genus *Blumenavia*?

Authors: We added a paragraph in the text (Results and discussion Molecular phylogeny) explaining the congruence with the geographic distribution and the other analysis adopted in this work to delimit the species in *Blumenavia*, this paragraph can be seen below:

Additional data that supports the three species delimitation, as well as in the case of the other species in *Blumenavia*, are regarding the geographical distribution (Fig 3). *Blumenavia angolensis* identified in the Southern and Northeastern Brazil are the new species *B. crucis-hellenicae* sp. nov. and *B. baturitensis* sp. nov., respectively. Both segrageted from *B. angolensis* from Africa. The type location of *Blumenavia rhacodes* is Southern Brazil, collection from this region maintains its identity and was shown to be distinct from others identified as *B. rhacodes* from Mexico; the latter is a novelty for science: *B. heroica* sp. nov. In addition, in Mexico, *B. toribiotalpaensis* was considered by other authors [10–13] as *B. rhacodes*, but both were shown to be distinct, assuming the invalidation of this synonymization. Given the restrict distribution found for all species in our revision on genus *Blumenavia*, our results rise the possibility for existing a phylogeographical pattern in *Blumenavia*, which need to be further explored in future studies with better sampling.

**Reviewer #1:** Line 107: “… size, color and texture of expanded basidiomata, receptacles, glebifers, volvae, as well as rhizomorphs were analyzed at a macroscopic level, therefore are written in the description based on fresh basidiomata.”

Most of the material was from herbarium, with dry basidiomata.

Authors: We showed the macroscopic characters based on collector description, because it can change when dry, e.g. the color becomes yellowish in all of them.

**Reviewer #1:** Line 109: *…exoperidia, mesoperidia, and endoperidia.*

Microscopic analyzes of the mesoperidia and endoperidia were not shown in the descriptions (they are not informative characters). However, this study is the first in which the different peridium layers have been analyzed in *Blumenavia*.

I suggest published as supplemental data. As well as all other new characters revealed but uninformative for delimitation of *Blumenavia* species. These characters are mentioned only at the conclusion.

Authors: The suggestion was accepted. We added a characters table in Supplementary Material. We modified the text as follows: “Since these characters were not informative to segregate species, microscopic analyzes of basal and apical arms, glebifers, basal exoperidia, mesoperidia and endoperidia are not included in taxonomical descriptions (see S1 Table). However, this study is the first in which the different peridium layers have been analyzed in *Blumenavia* (see layers detail in Fig 9A). In this paper, only the informative characters are included in the description of each species.”

**Reviewer #1:** Line 199: to change: 3,803 to 3,767. (3,537+139+91 = 3,767?)

Authors: The number was corrected in the text: 3,573 were constant.

**Reviewer #1:** Line 120: *In this paper, only the informative characters are included in the description of each species.*

Which are they? They were only mentioned in the abstract.

Authors: In the abstract only was included the macromorphological characters, the word morphological was modified to macromorphological to clarify this.

A section named **Morphological characters** was added before Taxonomy, to group informative and not informative characters. These characters have been cited only in conclusions and were transferred from this part to the new one.

**Reviewer #1:** Line 143, Table 1:

- LSU for *Blumenavia angolensis:* Line 285, mentions ITS

Authors: The mistake was corrected in the text, the table is correct.

- ICN 177268 and ICN 177269: Were not cited in the text.

Authors: The mistake was corrected in the text, the table is correct.

**Reviewer #1:** Line 194: discussion: The clades of the *Blumenavia* species need to be further discussed.

The figure 3 is not discussed in the text.

What does say the geophylogeny proposed for *Blumenavia*?

Authors: We added a paragraph in the text (Results and discussion; Molecular phylogeny) explaining the congruence with the geographic distribution and the other analysis adopted in this work to delimit the species in *Blumenavia,* as can see in question of line 84.

**Reviewer #1:** Line 212: to change: five to six

Authors: Not accepted, because we consider the five clades and the singleton, resulting six, the text was maintained the same.

**Reviewer #1:** Line 219 and 220: “As shown in Fig 1, *B. baturitensis* sp. nov., *B. heroica* sp. nov. and *B. rhacodes* grouped together in a clade, within three independent groups, but with low support values; and, since these species show few unremarkable morphological differences, …”

Can they be called semi-cryptic species?

Authors: Actually, it is preferable to not use the term semicryptic species, only cryptic species. Since we can find morphological characters, even inconspicuous, they are not cryptic anymore. It is possible find this information in Struck et al. 2017 and Martín et al. 2018.

Struck, Torsten & Feder, Jeffrey & Bendiksby, Mika & Birkeland, Siri & Cerca, José & Gusarov, Vladimir & Kistenich, Sonja & Larsson, Karl-Henrik & Liow, Lee Hsiang & Nowak, Michael & Stedje, Brita & Bachmann, Lutz & Dimitrov, Dimitar. (2017). Finding Evolutionary Processes Hidden in Cryptic Species. Trends in Ecology & Evolution. 33. 10.1016/j.tree.2017.11.007.

Martín, María & Zhang, L.-F & Fernández-López, Javier & Dueñas, Margarita & Rodríguez-Armas, J.L. & Tejera, Esperanza & Telleria, M.. (2018). Hyphoderma paramacaronesicum sp. nov. (Meruliaceae, Polyporales, Basidiomycota), a cryptic lineage to H. macaronesicum. Fungal Systematics and Evolution. 10.3114/fuse.2018.02.05.

**Reviewer #1:** Line 224: Fig. 3.

This figure is not discussed in the text.

It can be included in the discussion of the article.

What does say the geophylogeny proposed for *Blumenavia*?

Authors: We’ve added a paragraph in the text, as can see in the questions about lines 84 and 194.

**Reviewer #1:** Line 243: “ 2’. Glebifers occupying the external borders of the length of the arms ....................................... 3”

It needs to be the opposite.

Suggestion:

Glebifers as a wrinkled tentacular projections structure, or triangular, quadrangular or irregular points projections

Authors: Suggestion accepted.

**Reviewer #1:** Line 246 and 247: Add the apical exoperidium hyphae.

*Blumenavia heroica* sp. nov. is the only species in this genus with filamentous and subglobose to ellipsoid apical exoperidium hyphae.

The other species are filamentous and elongate hyphae or only filamentous.

Authors: Suggestion accepted.

**Reviewer #1:** Line 271: Why didn't you include the spore size in the description?

(3.2–)3.3–3.6 4.0 × (1.3–) 1.4–1.8(–1.9) μm

Authors: This information was added in the text.

**Reviewer #1:** Line 274: Scales above?

Authors: This term refers to colored parts of the upper part of the volva, it can be seen in Fig. 4A. Dring, 1980 [4] used this term to refer to this part too.

**Reviewer #1:** Line 316: “… (F, G) Torn and twisted glebifers in arms; …”

It is not clear in the photos.

Authors: As a little studied group, many terms that we had to add were new to describe the characters well, these are one.

**Reviewer #1:** Line 327: to add leg. before “…G.”. The same with A. of Line 356 and others.

Authors: The abbreviation leg. was added before these names, and in the others in all article.

**Reviewer #1:** Line 328-329: “(UFRN-Fungos 2868!, ITS nrDNA, LSU nrDNA, ATP6, RPB2 and TEF-1α GenBank sequences: MG817725, MG817733, MH061924, MH061933, MH061942).”

Suggestion: Respectively

or

ITS nrDNA sequence GenBank MG817725, LSU nrDNA sequence GenBank MG817733, ATP6 sequence GenBank MH061924, RPB2 sequence GenBank MH061933, TEF-1α sequence GenBank MH061942.

Authors: Respectively was added in the end of the phrase, and in the other species too.

**Reviewer #1:** Indicate in the figure with arrows.

It will make it easier to observe such detailed information.

- Line 340: Fig 5A–D:
- Line 400: Fig6A–C: “…marked longitudinal groove …”
- Line 468: Fig7: The best photo to see the longitudinal groove is the letter J
- Line469: Fig 7A–E: “…parallel lines curving inward forming triangular, quadrangular …”
- Line 470: Fig 7F–N: “…or parallel lines curving inward not sparse in the upper half of the basidiomata with triangular to quadrangular projections to the base…”
- Line 513: Fig 9H: “(H) Dry glebifer”

Authors: Suggestions accepted. The Figures 5, 6, 7 and 9 were replaced with the arrows.

**Reviewer #1:** Line 341-342: “…by the inner side of the arms and…”

Delete this part. The projections are already on the inner side of the arms.

Authors: Suggestion accepted.

**Reviewer #1:** Line 345: Are apical exoperidium hyphae informative character?

Isn't basal exoperidium hyphae informative character?

It is not clear in the text.

Authors: A section named **Morphological characters** was added before Taxonomy, to group informative and not informative characters.

**Reviewer #1:** Line 349-350; 478-479: Basidiospores ~~elongated to~~ cylindrical. Based on the work of Bas (1969), 2.42 and 2.35 characterizes only cylindrical spores. Elongated is 1.60–2.00.

Authors: Yes, these were a mistake, it was corrected in the text.

**Reviewer #1:** Line 349-350: “Comments. In Rodrigues and Baseia [9] collection UFRN-Fungos 1943 was identified as *B. angolensis* based on morphological characters alone. However, *B. baturitensis* sp. nov. differs from *B. angolensis* by having larger basidiomata (91–119 mm), yellowish and thick arms, tapering from the middle of the basidiomata to the apex (Fig 5A, B), and glebifers as wrinkled tentacular projections (Fig 5D). Trierveiler-Pereira et al. [10], also using only morphological characters, synonymized *B. baturitensis* sp. nov. with *B. rhacodes*. However, we observed that the absence of a groove in the outer face of the arms, and the glebifer shape separate *B. rhacodes* from *B. baturitensis* sp. nov.; as also confirmed by molecular data.”

Suggestion for this part of the paragraph:

Specimens of this new species (collection UFRN-Fungos 1943) were identified based on morphological characters alone in Rodrigues and Baseia (9) and Trierveiler-Pereira et al. [10] as *B. angolensis* and B rhacodes, respectively. However, *B. baturitensis* sp. nov. differs from *B. angolensis* by having larger basidiomata (91–119 mm), yellowish and thick arms, tapering from the middle of the basidiomata to the apex (Fig 5A, B), and glebifers as wrinkled tentacular projections (Fig 5D). While *B. rhacodes* has no groove in the outer face of the arms, and the glebifer shape different, as also confirmed by molecular data.

Authors: The text was added in the paper, but in the last phrase the correct information is: “*B. rhacodes* has grooves”

**Reviewer #1:** Line 380: to change: paratype: (K) to paratype; (K)

Authors: Not accepted, because the photos H to K refers to ICN 177268 and after the colon becomes the explanation of each. The order was replace to alphabetic order.

**Reviewer #1:** Line 434-437: “ Molecular analyses do not confirm the existence of a cluster with white colored species as mentioned by other authors [4,10] since *Blumenavia crucis-hellenicae* sp. nov. shares a common ancestor with *B. angolensis* and *B. toribiotalpaensis* (Fig 1).”

Authors: Dring, 1980 [4] synonymized *B. usambarensis* with *B. angolensis* because the white color of the both species and other characters, this treatment was adopted by Trierveiler-Pereira et al. 2014 [10], who identified the white species of Brazil as *B. angolensis* utilizing the color of the basidiomata with a parameter.

In this paragraph mentioned by the reviewer, we intend to show how white colored species have not been grouped phylogenetically, because white species *Blumenavia crucis-hellenicae* sp. nov. and *B. angolensis* share a common ancestor with *B. toribiotalpaensis*, a yellowish species. The paragraph was modified to improve the explanation of the idea: “Molecular analyses do not confirm the existence of a cluster with white colored species as mentioned by other authors [4,10] since the white species *Blumenavia crucis-hellenicae* sp. nov. and *B. angolensis* shares a common ancestor with *B. toribiotalpaensis*, one of the yellowish species (Fig 1).”

**Reviewer #1:** Line 443: Fig 7: Are all photos informative?

Authors: No, after a review we could replace the figure and take only the informative photos.

**Reviewer #1:** Line 492: What is the closest *Blumenavia* species to *B. heroica*?

The clades of the *Blumenavia* species need to be further discussed.

Authors: the closest *Blumenavia* species to *B. heroica* is *B. rhacodes* and *B. baturitensis* sp. nov. The text was modified, and this clade was better discussed after this review, like you can see in Results and discussion - Molecular phylogeny.

**Reviewer #1:** Line 497-498: “These characters, however, did not prove informative for a correct identification of the species.”

By other authors?

Authors: No, this information refers to specimens in *Blumenavia heroica.* We excluded this phrase in the comments, admitting that was confusing the explanation.

**Reviewer #1:** Line 497-498: “In the micromorphological analyses, however, these specimens share apical exoperidial hyphae with of the same pattern (filamentous or globose) and the same cylindrical basidiospore dimensions.”

Confused.

You are referring to:

1 - the collections cited by Calonge or,

2 - characters shared with other *Blumenavia* species

Authors: We are referring to *Blumenavia heroica* sp. nov.

These comments were changed to better explanation:

Calonge et al. [12] cited a number of collections under *Blumenavia rhacodes*: IBUG 240a, here designate as *B. toribiotalpaensis*, as well as those located in XAL herbarium (X. Madrigal 4535 E. Gándara 1455, R. Mede1 654, J. C. Anel 410, F Tapia 712, S. Chacón 5107, R. Medel 672, S. Chacón 5257-A, D. Jarvio 370, D. Jarvio 778, R. Farias, R. Medel 662, S. Chacón 4475), in which some of them here are considered the new species *B. heroica* sp. nov. *Blumenavia* *heroica* sp. nov. has a large macromorphological variety, with basidiomata with varying sizes (45–80 mm), besides presenting arms and glebifers with distinct forms. However, in the micromorphological analyses, these specimens analyzed here share apical exoperidial hyphae with of the same pattern (filamentous or globose) and the same cylindrical basidiospore dimensions. Confirming microscopic studies, phylogenetic analyses demonstrated that these specimens belong to a single well-supported clade (PP = 0.9). That clade shares a common ancestor with *B. rhacodes* and *B. baturitensis* sp. nov., all these three species have a yellowish basidioma, but *B. heroica* sp*.* nov. differs in the apical exoperidia hyphae and in the basidiomata height. *Blumenavia toribiotalpaensis* also differs from *B. heroica* sp. nov. in that characters mentioned for *B. rhacodes* and *B. baturitensis* sp. nov., and in the glebifer shape.

**Reviewer #1:** Line 513-514: to change: (J-K) to (J-M)

Authors: Not accepted, because only the photos J and K refer to ICN 177266, the photo L refers to specimen PACA12552 and M to PACA12550, these was indicating in the legend, as you can see: “(L) Rhizomorph hyphae in 1% Congo red, PACA 12552. (M) Basidiospores in 5% KOH, PACA 12550.”

**Reviewer #1:** Line 517: to change: I by to send by.

Authors: Partially accepted, the letter I was maintained because refers to the photo I, also sent by Fazolino E.P. We changed in the legend: (A) bar = 0.2 mm; (B, C, E) bar = 10 mm; (F–I) bar = 1mm; (J–M) bar = 5 µm. Photographs: (B, I) sent by Fazolino E. P.; (D) sent by Matthias Schultz. All legends were changed to this format.

**Reviewer #1:** Line 644: Remove italics from “glebifers”

Authors: All characters were in italic in all descriptions, all these italicizations were removed.

**Reviewer #1:** Line 653: Excellent that you have analyzed the type species.

The only way to differentiate *B. usambarensis* from *B. angolensis* is by mycoscopic characters?

Are the rhizomorphs of the B 700016468 collection preserved? Is there no possibility that fragments came from another part of the basidiomata?

Authors: Unfortunately, the only way is by microscopic characters.

Yes, the rhizomorphs of the B 700016468 (*B. usambarensis*, holotype) collection were preserved. All analysis were made from all parts of the basidiomata, and the differences were found in basidiospores and in rhizomorphs. If analyzed rhizomorph fragments became from another part of the basidiomata it should match with the other parts description, but it is not the case, and, as said above, the exsiccate is well preserved.

**Reviewer #1:** Line 701: Before references: Author Contributions

Authors: This information were added in the text.

**Reviewer #2:** The authors present a highly interesting topic for the criptic taxa of the genus Blumenavia, combining morphological and molecular data of the analyzed species, their ecology, as well as comparative revision of the material form collections, especially type species.

The presented data are sufficient, representative and well documented, presenting new and original information. The interpretations are logical and well supported.

The text is concise and clearly explains topics, methodologies, data and conclusions.

The title of the paper clearly characterizes its content.

The abstract is clear and informative and comprise adequately the content of the manuscript.

The applied methods are appropriate, presented clearly and understandable.

The descriptions of species are fairly well performed and relevant.

Figures and tables are clearly presented and correctly labeled. They are instructive and captions unambiguously explain

pictured details. Important features from all available material are presented with clear photos. Additionally, including the reprints of the old collections, representing all data at the same place, is a very good way for comparison.

All the references are adequate and necessary, considering recent research.

Conclusions are highly supported by the presented data and the results.

**Reviewer #2 Minor issues:**

p. 9 Molecular phylogeny

The number of the specimens and the number of the sequences written in the text is not the same as shown in Table 1.

Please check it.

Authors: Thank you for the observation, we replace the wrong information in the text, the table was corrected.

p. 14, 322 *B. heroica* should be changed with *B. crucis-hellenicae*. (*B. heroica* belongs to the white colored species and *B. crucis-hellenicae* is with yellowish color, as it is shown in all other parts for both species – key, description, photos).

Authors: We don’t agree with this alteration because *B. heroica* isn’t a white colored specie, but yellowish one, as seen in its description p.20, line 465 (p.23, lines 544-545, in revision version): “Receptacle 3 to 4 arms united above, free in the base, whitish yellow to pale yellow”. In contrast, *B. crucis-hellenicae* is a white specie, seen in p. 17, line 398 (p. 20, lines 475-476, in revision version): “Receptacle 3 to 4 arms united above, free in the base, white”.

**Reviewer #3:** Dear Authors,

Thank you for the opportunity to review a nice taxonomic manuscript. It would be more traditional to see it in Mycologia or Taxon magazines, but regarding novel adds to the (still) not quite traditional methods to delimit species boundaries like

phylogeny PLoS definitely can serve as a proper place for it. I feel confident to recommend your manuscript to be published with minor revisions. Also, I have a few questions, below.

1. I believe Table 1 better belongs to the Supplements.

Authors: According to other papers in the same line, this tables are important to inform the material analyzed and summarize the Genbank numbers, we maintained in the text.

**Reviewer #3**: Material and Methods

Phylogenetic Analysis

2. Did you obtained all sequences of the specimens from genus Blumenavia you have used for your reconstructions? Have you compared your sequences to the available ones in GenBanks and how you find their identity/ similarity?

Authors: Yes, the sequences from *Blumenavia* genus used in the reconstruction was obtained by us, except *B. angolensis*. In GenBank only more one sequence was available, ATP6 from *B. rhacodes: Blumenavia rhacodes* voucher LTP230 (ICN) ATP synthase subunit 6 (atp6) gene, partial cds; mitochondrial. GenBank: KF783253.1, it corresponds to ICN 176968, this exsiccate was revised for us and we made the sequencing again (ITS nrDNA, LSU nrDNA, ATP6 and TEF-1α GenBank sequences: MG817718, MG817729, MH061922, MH061936), because of that didn’t use this.

As a revision work the aim was determinate the differences within the species of this genus, we’ve got all exsiccates available for loan in order morphological and molecular comparisons.

**Reviewer #3:** Results and Discussion

Molecular phylogeny, lines 196-198

3. For this study, DNA sequences of 12 specimens of *Blumenavia* were obtained: 9 ITS, 8 LSU, 6 ATP6, 9 RPB2 and 9 TEF-1a (Table 1). Sequences from *B. usambarensis* were not obtained.

better to modify

For this study, DNA sequences of 12 specimens of *Blumenavia* were obtained: 9 ITS, 8 LSU, 6 ATP6, 9 RPB2 and 9 TEF-1a (Table 1), except *B. usambarensis*.

Authors: This suggestion was accepted and was corrected in the manuscript.

**Reviewer #3:** 4. Did all trees (MP, ML and BI) have the same topology?

Authors: These topologies might see in TreeBase: http://purl.org/phylo/treebase/phylows/study/TB2:S2593; for reviewer access: http://purl.org/phylo/treebase/phylows/study/TB2:S25096?x-access-code=cb0af291cea482c1bfa56649485eb9b2&format=html, and for facility the explanation was pasted below with MP, ML and BI, respectively.

The tree analysis maintained the same five clades and the singleton with *Blumenavia angolensis.* The text below was added in Results and discussion to better explanation of this differences:

MP, ML and BI show *B. baturitensis* sp. nov., *B. heroica* sp. nov. and *B. rhacodes* grouped together in a clade, but in ML *B. angolensis* is grouped in the middle with *B. rhacodes* with low support. In MP *Blumenavia angolensis* forms a singleton in a bigger clade of *B. baturitensis*, *B. rhacodes* and *B. heroica*, and in BI as a singleton together with the clades *B. toribiotalpaensis* and *B. crucis-hellenicae.* An explanation is that *B. angolensis* has only LSU sequence, in contrast with the others.

*Blumenavia toribiotalpaensis* and *B. crucis-hellenicae* are grouped in a clade in BI and ML, in BI this clade shares a common ancestor with *B. angolensis*, as mentioned before. In MP *B. crucis-hellenicae* is a sister group of the clade with *B. angolensis* and the one with *B. baturitensis*, *B. rhacodes* and *B. heroica*, *B. toribiotalpaensis* in this analysis appears out of all these clades.

An evolutionary relationship for *B. angolensis* may be unclear because the lack of some markers, but these differences between the trees don’t reveal an incongruency in the separation of the species. Defining infrageneric relationship was not intended in this work, so evolutionary placement of *B. angolensis* can made in future works with better sampling of this species.

**Reviewer #3:** *Blumenavia angolensis* (Welw. & Curr.) Dring, Kew Bull. 35(1): 53 (1980), description

5. I believe, "covered with glebal mass." in line 279 has to be removed.

Authors: Yes, thank you for the observation, it was removed.

**Reviewer #3:** 6. Cannot access the alignments at http://purl.org/phylo/treebase/phylows/study/TB2:S25096?x-access192

code=cb0af291cea482c1bfa56649485eb9b2&format=html

Authors: Sorry about this inconvenient, we made a resubmission, the new address is: http://purl.org/phylo/treebase/phylows/study/TB2:S25936?x-access-code=a21d01f584a54a1997c0c42350b6f8f1&format=html
